# Molecular Typing and Phenotype Characterization of Methicillin-Resistant *Staphylococcus aureus* Isolates from Blood in Taiwan

**DOI:** 10.1371/journal.pone.0030394

**Published:** 2012-01-23

**Authors:** Wei-Yao Wang, Tzong-Shi Chiueh, Jun-Ren Sun, Shin-Ming Tsao, Jang-Jih Lu

**Affiliations:** 1 Graduate Institute of Medical Science, National Defense Medical Center and Tri-Service General Hospital, Taipei, Taiwan, Republic of China; 2 Division of Clinical Pathology, Department of Pathology, National Defense Medical Center and Tri-Service General Hospital, Taipei, Taiwan, Republic of China; 3 Division of Infectious Disease, Fong-Yuan Hospital, Taichung, Taiwan, Republic of China; 4 Division of Clinical Microbiology and Department of Internal Medicine, Chung Shan Medical University Hospital, Taichung, Taiwan, Republic of China; 5 Graduate Institute of Clinical Medical Science, China Medical University, Taichung, Taiwan, Republic of China; 6 Department of Laboratory Medicine, Linkou Chang-Gung Memorial Hospital, Taoyuan, Taiwan, Republic of China; National Institutes of Health, United States of America

## Abstract

**Background:**

*Staphylococcus aureus* causes a variety of severe infections such as bacteremia and sepsis. At present, 60–80% of *S. aureus* isolates from Taiwan are methicillin resistant (MRSA). It has been shown that certain MRSA clones circulate worldwide. The goals of this study were to identify MRSA clones in Taiwan and to correlate the molecular types of isolates with their phenotypes.

**Methods:**

A total of 157 MRSA isolates from bacteremic patients were collected from nine medical centers. They were typed based on polymorphisms in *agr*, SCC*mec*, MLST, *spa*, and *dru*. Phenotypes characterized included Panton-Valentine leucocidin (*pvl*), inducible macrolide-lincosamide-streptogramin B resistance (MLSBi), vancomycin (VA) and daptomycin (DAP) minimal inhibitory concentrations (MIC), and superantigenic toxin gene profiles. Difference between two consecutive samples was determined by Mann-Whitney-U test, and difference between two categorical variables was determined by Fisher's exact test.

**Results:**

Four major MRSA clone complexes CC1, CC5, CC8, and CC59 were found, including 4 CC1, 9 CC5, 111 CC8, and 28 CC59 isolates. These clones had the following molecular types: CC1: SCC*mec*IV and ST573; CC5: SCC*mec*II and ST5; CC8: SCC*mec*III, ST239, and ST241, and CC59: SCC*mec*IV, SCC*mec*V_T_, ST59, and ST338. The toxin gene profiles of these clones were CC1: *sec*-*seg*-(*sei*)-*sell*-*sel*m-(*seln*)-*sel*o; CC5: *sec*-*seg*-*sei*-*sell*-*sel*m-(*seln*)-*selp*-*tst1*; CC8: *sea*-*selk*-*selq*, and CC59: *seb*-*selk*-*selq*. Most isolates with SCC*mec*V_T_, ST59, *spa*t437, and *dru*11 types were *pvl* ^+^ (13 isolates), while multidrug resistance (≥4 antimicrobials) were associated with SCC*mec*III, ST239, *spa* t037, *agr*I, and *dru*14 (119 isolates) (*p*<0.001). One hundred and twenty four isolates with the following molecular types had higher VA MIC: SCC*mec*II and SCC*mec*III; ST5, ST239, and ST241; *spa* t002, t037, and t421; *dru*4, *dru*10, *dru*12, *dru*13, and *dru*14 (*p*<0.05). No particular molecular types were found to be associated with MLSBi phenotype.

**Conclusions:**

Four major MRSA clone complexes were found in Taiwan. Further studies are needed to delineate the evolution of MRSA isolates.

## Introduction

*Staphylococcus aureus* is one of the most common pathogens in both community- and hospital-associated infections [1], [2], [3]. It is also the leading cause of skin, [4], [5], cardiovascular tissue [6] and osteoarticular tissue [7] infections, pneumonia [5], [8], and bacteremia [9]. Methicillin-resistant *S. aureus* (MRSA) has emerged in the 1960s and reached a prevalence of 60–80% in the 1980s [1], [10], [11]. Patients with invasive MRSA infections have a higher mortality rate than those with noninvasive MRSA infections [9], [11], [12]. MRSA is also the leading cause of death due to microbial infections [4], [11].

Several molecular methods have been developed to type MRSA isolates, including pulsed-field gel electrophoresis (PFGE) [13], multilocus sequence typing (MLST) [14], and typing based on polymorphisms of the following genetic loci: the staphylococcal cassette chromosome *mecA* (SCC*mec*) [15], the X region encoding protein A (*spa*) [16], the *mec*-associated hypervariable region (*dru*) [17], and the accessory gene regulator (*agr*) [18]. With these typing methods, some MRSA isolates including the New York/Japan (SCC*mec*II-ST5-*agr*II), Hungarian (SCC*mec*III-ST239-*agr*I), and USA 300 (SCC*mec*IV-ST8-*agr*I) clones have been identified worldwide [19], [20]. MRSA isolates also harbor a number of virulence factors including antibiotic resistance [21], [22], Panton-Valentine leukocidin [23], and exotoxins [19], [24].

Although MRSA typing is commonly done, few studies have been conducted to correlate genotypes with phenotypes. Furthermore, most MRSA typing performed in Taiwan are limited to isolates from one hospital [25], [26], [27]. In this study, we expanded our scope to include MRSA isolates from various locations in Taiwan. We also correlated molecular types of the MRSA isolates with their phenotypes based on the virulence factors present in each isolate.

## Materials and Methods

### Identification and determination of antimicrobial susceptibility of MRSA isolates

A total of 1,000 isolates suspected to be MRSA from various specimens were collected from March to August, 2003 as part of the Surveillance of Multicenter Antimicrobial Resistance in Taiwan (SMART) program. Ten hospitals of various medical centers participated in this program including three each in northern (hospitals N1, N2, N3), central (hospitals C1, C2, C3), and southern (hospitals S1, S2, S3) and one in eastern (hospital E1) part of Taiwan. *S. aureus* was identified as Gram-positive cocci with β-hemolysis on 5% sheep blood agar and giving positive results for catalase, coagulase, DNase, and mannitol fermentation tests. MRSA isolates were screened with 30-*u*g cefoxitin disc [28], [29] and confirmed to harbor the *mecA* gene by polymerase chain reaction (PCR) [15] and by oxacillin resistance [29]. Among the 1,000 isolates, 157 non-duplicated MRSA isolates from blood samples from 9 medical centers in Taiwan were used in this study, including 70 from N1, 4 from N2, 17 from N3, 3 from C1, 1 from C2, and 18 from C3, 24 from S1, 1 from S3, and 19 from E1 hospitals. Isolates that were resistant to four or more of the following antibiotics by the disc diffusion test were considered as multidrug resistance: gentamicin, trimethoprim/sulfamethoxazole (TMP-SXT), ciprofloxacin, erythromycin, clindamycin, ampicillin/sulbactam, ciprofloxacin, oxacillin, vancomycin, and teicoplanin. Inducible macrolide-lincosamide-streptogramin B resistance (MLSBi) was determined by the D-test, in which the erythromycin and clindamycin discs were placed 15 mm apart on blood agar plates [28]. The vancomycin (VA) MICs of the isolates were determined by both the agar dilution test [28] and the E-test with strips containing gradients of VA and daptomycin (DAP) according to manufacturer's instructions (AB BIODISK, AB bioMerieux, Sweden).

### Bacterial DNA extraction and SCC*mec* classification

Genomic DNA was extracted from each MRSA isolate using the Genomic DNA Mini Kit (Geneaid, Taiwan). The genes encoding the cassette chromosome recombinase (*ccr*) complex and the *mec* complex were typed by multiplex PCR (M-PCR) with 14 primers as described by Kondo et al. [15]. SCC*mec* types I to V were identified by comparing the M-PCR banding patterns of the isolates to those of the following reference strains: ATCC 10442 (SCC*mec* type I), N315 (SCC*mec* type II), 85/2082 (SCC*mec* type III), MW2 (SCC*mec* type IVa), WIS (SCC*mec* type V), and TSGH-17 (SCC*mec* type V_T_). Isolates initially determined to be SCC*mec*V were further analyzed with specific primers (*ccrC*-FR) for SCC*mec*V_T_ [30], which was recently renamed as SCC*mec*VII (the Taiwan clone) [31].

### Multilocus Sequence Typing (MLST)

The following seven housekeeping genes of each MRSA isolate were amplified and sequenced as described previously [14]: the carbamate kinase (arcC), shikimate dehydrogenase (*aroE*), glycerol kinase (*glp*), guanylate kinase (*gmk*), phosphate acetyltransferase (*pta*), triosephosphate isomerase (*tpi*), and acetyl coenzyme A acetyltransferase (*yqiL*) genes. The allelic number and profile (sequence type or ST) of each gene were determined by comparing the sequences to those of the known alleles deposited in the *S. aureus* MLST database (http://saureus.mlst.net/).

### Polymorphism of the X region encoding protein A (*spa* typing)

The X region of the *spa* gene contains variable numbers of 21- to 27-bp repeats, with the 24-bp repeat being most common [16]. The X region of each MRSA isolate was amplified by PCR as described by Shopsin et al. [32]. The amplified products were sequenced and analyzed by the Ridom StaphType software program (version 1.4; Ridom, GmbH, Wurzburg, Germany [http://spa.ridom.de/index.shtml]), which automatically determined the repeat profile and the *spa* type of each isolate [33].

### Polymorphism of the mec-associated hypervariable region

Amplification of the hypervariable region, which contains variable numbers of 40-bp direct repeat units (*dru*), was performed by PCR with primers orf145 and IS431mec as described by Senna et al. [17]. The copy number of *dru* in each MRSA isolate was determined by the size of the amplified fragment according to the following equation: *dru* copy no. = (size of PCR product−171)÷40 [17].

### Accessory gene regulator (*agr*) typing

The *agr* gene of each MRSA isolate was amplified with primers Pan, agr1, agr2, agr3, and agr4 as described by Gilot et al [18]. These primers allowed amplification of a 441-bp DNA fragment from *agr* group I, a 575-bp fragment from *agr* group II, a 323-bp fragment from *agr* group III, and a 659-bp fragment from *agr* group IV strains.

### Gene encoding Panton-Valentine leukocidin (*pvl*)

Amplification of the *pvl* gene was done with primers luk-PV-1 and luk-PV-2 as described by Lina et al. [23]. The reference strain TSGH-17 was used as the positive control, and the strain 85/2082 was used as the negative control.

### Superantigenic toxin gene profile

Each MRSA isolate was examined for the existence of a total of 18 genes encoding classical (*sea*, *seb*, *sec*, *sed*, *see*, *seg*, *seh*, *sei*, *selj*, *selk*, *sell*, *selm*, *seln*, *selo*, *selp*, *selq*, and *selr*) and newly described superantigenic (*tsst-1*) toxins by four multiplex PCRs, with *femA* and *femB* as positive control genes in the M-PCR [34].

### Discriminatory power of molecular typing systems and statistic methods

The discriminatory power of each typing method was determined by the Simpson's index of diversity as described by Hunter [35]. The difference between two consecutive samples within the same population was determined by the Mann-Whitney-U test if the population was not normally distributed (nonparametric), and the difference between two categorical variables was determined by the Fisher's exact test. A *p* value less than 0.05 was considered significant. The Pearson's test was used to determine whether two consecutive samples had a close (*r*≥0.7) or moderate (0.7>*r*≥0.4) correlation.

## Results

### MRSA identification and antimicrobial susceptibility determination

**Figure 1:**
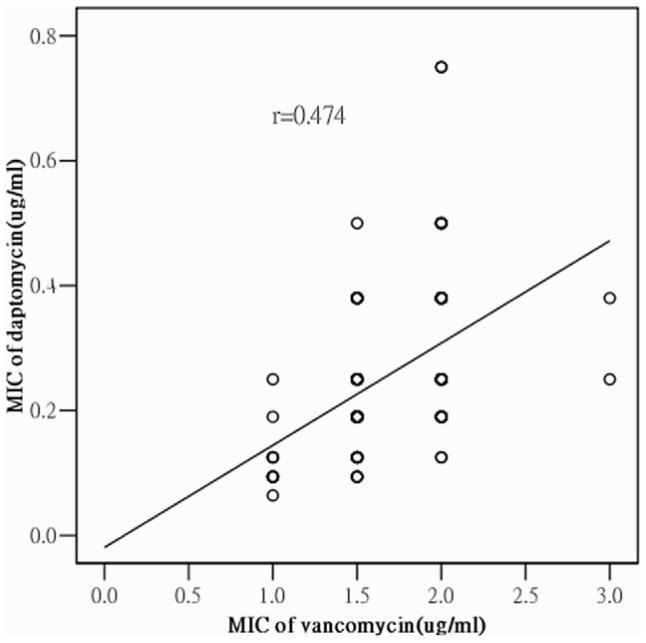
Correlation between vancomycin and daptomycin MICs of 157 MRSA blood isolates determined by E-test; *r* (Pearson's correlation coefficient) = 0.474.

Antimicrobial susceptibility testing by the disc diffusion method revealed that 119 (75.8%) of the 157 MRSA isolates were resistant to multiple drugs including erythromycin (149 isolates, 94.9%), clindamycin (136 isolates, 86.5%), TMP-SXT (113 isolates, 71.8%), and gentamicin (123 isolates, 78.2%) (Table 1). The mean vancomycin MIC was determined to be 1.56 mg/L (range:1–3 mg/L) by the agar dilution method (Table 1); this value was very close to that determined by the E-test (mean: 1.66 mg/L; range: 1–3 mg/L). The mean daptomycin MIC determined by the E-test was 0.25 mg/L (range: 0.064–0.75 mg/L). The Pearson's coefficient for correlation between vancomycin and daptomycin resistance showed a moderate correlation (*r* = 0.47) (Fig. 1).

**Table 1:** Correlations between molecular types and phenotypes of MRSA blood isolates.

Molecular types		Isolate number (percent)			Minimal inhibitory concentration (MIC) (mg/L)		
		Positive *pvl*	Positive D-test	Multidrug resistance^1^	VA (agar dilution)	VA (E-test)	DPC (E-test)
SCC*mec*	II	0(0)	0(0)	7(4.5)	1.61**	1.57	0.30*
	III	0(0)	12(7.6)	108(68.8)*	1.66*	1.74*	0.28*
	IV	2(1.3)	1(0.6)	3(1.9)	1.24	1.48	0.16
	V	0(0)	0(0)	1(0.6)	1.5	2.0	0.19
	V_T_	11(7.0)*	0(0)	0(0)	1.09	1.36	0.15
*agr*	I	13(8.3)	12(7.6)	114(72.6)*	1.56	1.67	0.26
	II	0(0)	1(0.6)	5(3.2)	1.45	1.60	0.22
MLST	ST5	0(0)	0(0)	7(4.5)	1.61**	1.72***	0.31*
	ST239	0(0)	10(6.4)	94(59.9)*	1.68*	1.75*	0.29*
	ST241	0(0)	2(1.3)	10(6.4)	1.42***	1.58	0.23
	ST59	12(7.6)*	0(0)	3(1.9)	1.17	1.39	0.15
	other	1(0.6)	1(0.6)	5(3.2)	1.55	1.65	0.17
*spa*	t002	0(0)	0(0)	6(3.8)	1.63**	1.75	0.32*
	t037	0(0)	11(7)	99(63.1)*	1.65*	1.72*	0.28**
	t421	0(0)	1(0.6)	7(4.5)	1.71**	1.78**	0.23***
	t437	11(7.0)*	0(0)	2(1.3)	1.18	1.42	0.15
	other	2(1.3)	1(0.6)	5(3.2)	1.30	1.47	0.16
copy no. of *dru*	4	0(0)	0(0)	8(5.1)	1.60**	1.70	0.29*
	6	0(0)	2(1.3)	6(3.8)	1.44	1.69	0.24
	9	4(2.5)	1(0.6)	5(3.2)	1.22	1.43	0.14
	10	0(0)	1(0.6)	5(3.2)	1.50**	1.64	0.22**
	11	8(5.1)*	0(0)	3(1.9)	1.36	1.50	0.17
	12	1(0.6)	0(0)	9(5.7)	1.59**	1.73**	0.23*
	13	0(0)	0(0)	5(3.2)	1.60***	1.80**	0.25**
	14	0(0)	9(5.7)	74(47.1)*	1.71*	1.76*	0.30*
	other	0(0)	0(0)	4(2.5)	1.33	1.42*	0.21
Total No. (%) (or mean for MIC)		13(8.3)	13(8.3)	119(75.8)	1.56	1.66	0.25

*p<0.05,

**p<0.01,

***p<0.001.

Abbreviations: VA, vancomycin; DPC, daptomycin; *pvl*, Panton-Valentine leucocidin; *agr*, accessory gene regulator.

1 Multidrug resistance: resistance to ≥4 classes of antibiotics (disc diffusion test).

### Molecular typing

There were 5 SCC*mec* types including types II (9, 5.7%), III (115, 73.2%), IV (21, 13.4%), V (1, 0.6%), and V_T_ (11, 7.0%) (Table 2). Only two *agr* groups were found, and most (147, 93.6%) isolates belonged to *agr* group I. A total of 7 MLST types were identified, including ST5 (9, 5.7%), ST239 (99, 63.0%), ST241 (12, 7.6%), ST59 (27, 17.2%), and others [ST338 (1, 0.6%), ST573 (4, 2.5%), and ST900 (2, 1.3%)]. Two new single locus variants of ST241 were identified in 3 isolates (Table 2). For *spa* typing, a total of 14 *spa* types were found; the following 4 types were more prevalent: t037 (107, 68.2%), t437 (20, 12.7%), t002 (8, 5.1%), and t421 (7, 4.5%). There were 11 *dru* types determined with *dru*14 (76, 48.4%), *dru*9 (23, 14.6%), *dru*11 (11, 7.0%), *dru*12 (11, 7.0%), *dru*4 (10, 6.4%), *dru*6 (8, 5.1%), and *dru*10 (7, 4.5%) being more common. The discrimination indices, defined as Simpson's diversity index (D value), for SCC*mec*, *agr*, MLST, *spa*, and *dru* typing were 0.713, 0.232, 0.842, 0.785, and 0.945, respectively.

**Table 2:** Clonal complexes and relationship between various molecular types of 157 MRSA blood isolates.

Clonal complex (CC)	MLST^1^	*agr* group	SCC*mec* type	*spa* type	No.
CC8	ST239	I	III	t037	92
				t421	4
				t138	1
				t388	1
				t3519	1
	ST241	I	III	t037	9
				t421	3
CC5	ST5	I	II	t002	3
		II	II	t002	5
				t242	1
CC59	ST59	I	IV	t437	11
				t1751	2
				t3592	3
		II	IV	t084	1
		I	V_T_	t437	9
				t3592	1
	ST338	I	V_T_	new^4^	1
CC1	ST573	I	IV	t3406	1
		II	IV	t3406	1
				t037	1
				t3525	1
Others	ST900	I	III	t037	2
	new^2^	I	III	t037	2
	new^3^	I	V	t037	1

1 MLST allelic profile in the order of *arcC*-*aroE*-*gypF*-*gmk*-*pta*-*tpi*-*yqiL*;

2 the alleic profile of 2-3-1-1-new-4-30;

3 the alleic profile of 2-3-1-1-4-99-30 after comparing the sequences and allelic profiles of with those deposited in the *S. aureus* MLST database (http://saureus.mlst.net/);

4 new *spa* type (alleic profile: 4-20-17-16- 34) after comparing the sequences and allelic profiles with those deposited in the *spa* typing website (http://spa.ridom.de/index.shtml).

The distribution of various molecular types of 157 MRSA blood isolates among 9 medical centers in Taiwan was shown in Table S2. Although it seemed that most identified molecular types were found equally in four areas of Taiwan, the majority of isolates with ST59, SCC*mec*IV and V_T_, and *spa* t421 were recovered in N1 hospital. All four ST573 and two ST900 isolates were recovered from the same hospital (N1) in northern Taiwan (Table S2).

Significant association was noted between various molecular types (Table S1). For example, SCC*mec*II isolates were distributed among *agr* group II, ST5, *spa* t002 and *dru*4 types (*p*<0.001). Similarly, SCC*mec*III isolates were mostly found in *agr* group I, ST239, ST241, *spa* t037, and *dru*14 types (*p*<0.05 for ST241 and *p*<0.001 for other corresponding types). SCC*mec*IV isolates were mainly seen in *agr* group II, ST59, ST573, *spa* t437, and *dru*9 types (*p*<0.05 for *agr* group II and *p*<0.001 for other corresponding types), and SCC*mec*V_T_ isolates were mostly found in ST59, *spa* t437, and *dru*11 types (*p*<0.001).

With SCC*mec* and MLST combination typing, the MRSA isolates were classified into four clone complexes (CC), including CC1, CC5, CC8, and CC59 (Table 2). CC1 included SCC*mec*IV-ST573 (4 isolates, 2.5%). CC5 contained SCC*mec*II- ST5 (9 isolates, 5.7%). CC8 (111 isolates, 70.7%) comprised SCC*mec*III-ST239 (99 isolates, 63.1%) or ST241 (12 isolates, 7.6%). CC59 (28 isolates, 17.8%) consisted of SCC*mec*IV (17 isolates, 10.8%) or V_T_ (10 isolates, 6.4%)-ST59 (27 isolates, 17.2%) or ST338 (1 isolate, 0.6%) (Table 2). Five isolates did not belong to any of these 4 clone complexes.

### Correlation between molecular types and phenotypes

Thirteen (8.3%) of the 157 MRSA isolates were found to harbor the *pvl* gene (Table 1). These isolates were mostly distributed in SCC*mec*V_T_, ST59, *spa* t437, and *dru*11 types (*p*<0.001). The MLSBi phenotype was also present in 13 isolates (8.3%), but no significant association between MLSBi and any specific molecular type was found. The mutidrug resistance (≧4 classes of antimicrobials, including oxacillin) phenotype was significantly correlated with SCC*mec*III, *agr* group I, ST239, *spa* t037, and *dru*14 types (*p*<0.001). Isolates with higher vancomycin MIC were noted in SCC*mec*II and SCC*mec*III; ST5, ST239, and ST241; *spa* t002, t037, t421; *dru*4, *dru*10, *dru*12, *dru*13, and *dru*14 types (*p* value from <0.001 to <0.05 respectively), and those with elevated daptomycin MIC were found in SCC*mec*II and SCC*mec*III; ST5 and ST239; *spa* t002, t037 and t421; *dru*4, *dru*10, *dru*12, *dru*13, and *dru*14 types(*p* value from <0.001 to <0.05 respectively) (Table 1).

The most prevalent toxin genes detected by M-PCR were *selk* (134, 85%), *selq* (133, 85%), *sea* (105, 67%), and *seb* (26, 17%). Some toxin genes were associated with specific molecular types (Table S3). For example, *selk* and *selq* are associated with SCC*mec*III, SCC*mec*IV, and SCC*mec*V_T_; *agr* group I; ST239; *spa* t037 and t3592; and *dru* 14. The *seb* gene was mainly found in isolates with SCC*mec*IV and SCC*mec*V_T_, ST59, *spa* t437, *dru*9 and *dru*11 types. The *tst-1* gene was found to be associated with SCC*mec*II; *agr* group II; ST5; *spa* t002; and *dru*4 types (*p* value from <0.001 to <0.05 respectively) (Table S3). A total of 15 superantigenic toxin gene profiles were seen. The most prevalent ones were *sea*-*selk*-*selq* (95, 60.5%), *seb*-*selk*-*selq* (17, 10.8%), and *selk*-*selq* (12, 7.6%). One isolate had no detectable toxin gene (Table 3).

**Table 3:** Association of superantigenic toxin gene profiles with different SCC*mec* types and *agr* groups of MRSA blood isolates.

Superantigenic toxin gene profiles	SCC*mec* type	*agr* type	No. (%)
None	V_T_	I	3 (1.9)
*selk*-*selq*	III	I	12 (7.6)
*sed*-*seg*-*sei*-*sell*-*selm*-*sel) n*-*selo*-*selp*-*tst1*	II	II	1 (0.6)
*sec*-*seg*-*sell*-*selm*-*seln*-*selo*	IV	I	1 (0.6)
*sec*-*seg*-*sei*-*sell*-*selm*-*selo*	IV	II	2 (1.3)
*sec*-*seg*-*sei*-*sell*-*selm*-*selo*-*selp*-*tst1*	II	II	3 (1.9)
*sec*-*seg*-*sei*-*sell*-*selm*-*selo*-*selp*-*tst1*	II	I	1 (0.6)
*sec*-*seg*-*sei*-*sell*-*selm*-*seln*-*selo*	IV	II	1 (0.6)
*sec*-*seg*-*sei*-*sell*-*selm*-*seln*-*selo*-*selp*-*tst1*	II	I	1 (0.6)
*sec*-*seg*-*sei*-*sell*-*selm*-*seln*-*selo*-*selp*-*tst1*	II	II	1 (0.6)
*sec*-*seg*-*sei*-*sell*-*selm*-*seln*-*selo*-*selp*-*tst1*	IV	I	1 (0.6)
*seb*-*selk*-*selq*	IV	I	8 (5.1)
*seb*-*selk*-*selq*	V_T_	I	8 (5.1)
*seb*-*selk*-*selq*	IV	II	1 (0.6)
*seb*-*selk*-*selp*-*selq*	IV	I	6 (3.8)
*seb*-*sei-selk*-*selq*	IV	I	2 (1.3)
*sea*	III	I	7 (4.5)
*sea*-*selk*-*selq*	III	I	95 (60.5)
*sea*-*selk*-*sell*	V	I	1 (0.6)
*sea*-*sec*-*seg*-*sei*-*sell*-*selm*-*selo*-*selp*-*tst1*	IV	II	1 (0.6)
*sea*-*seb*-*selk*-*selq*	III	I	1 (0.6)
Total			157 (100)

## Discussion

MRSA strains were originally found in hospital settings (HA-MRSA) in the 1960s [3], [8], but community-associated MRSA (CA-MRSA) strains have emerged in recent 20 years [36], [37]. The prevalence of MRSA in United States is shown to be increased from 22.8% to 56.2% during a 11-year surveillance study by the Center for Disease Control and Prevention [1]. The reports of the Taiwan Surveillance of Antimicrobial Resistance I and II (TSAR I and II) in 2000 indicate that 60% of the 400 *S. aureus* isolates from 65 hospitals in Taiwan were MRSA [10]. The mortality rate of patients with MRSA bacteremia has been shown to be as high as 33% [9], [27]. Because of the great impact of MRSA on patients' outcome, we investigated the association between genotypes and phenotypes of MRSA isolates from patients with bacteremia. Among the 157 independent blood MRSA isolates from 9 medical centers in Taiwan, 124 (79%) were HA-MRSA (SCC*mec*II and SCC*mec*III) and 33 isolates (21%) were CA-MRSA (SCC*mec*IV, SCC*mec*V, and SCC*mec*V_T_). Most isolates (152, 96.8%) were found to belong to the following four clonal complexes: CC1, CC5, CC8, and CC59 (Table 2). The SCC*mec*III-ST239 isolates were found to have a higher rate of multidrug resistance than other isolates, a finding similar to those of previous reports [5], [38]. Interestingly, we found strains with particular molecular types such as ST573 and ST900 were exclusively recovered from one university-affiliated teaching hospital in northern Taiwan (N1), and the ratios of molecularly CA-MRSA stains (ST59, SCC*mec*IV and V_T_, and *spa* t421) were higher than those recovered from other hospitals (Table S2). These findings showed that the molecular types of MRSA blood isolates were not equally distributed in the island and the prevalence CA-MRSA might increase in some hospitals, which was compatible with the previous reports [39], [40].

A pandemic MRSA lineage, ST239, has been found to descend from ST8 and ST30 parents (both belonging to CC8) through simple chromosome replacement instead of movement of mobile genetic elements [41]. There is only one allelic difference (*arcC*) in 7 housekeeping genes used for MLST between ST8 and ST239, but they are distinguished at least 6 surface protein-encoding genes including *spa*. Recently, Harris et al had delineated the intercontinental spread and microevolution of the MRSA ST239 isolates by mapping genome-wide single-nucleotide polymorphisms (SNPs) to reference sequence [42]. They found MRSA ST239-*spa* t037, responsible for the South American clade and scattering isolates from Europe and Asia, represented the ancestral ST239 *spa* type. MRSA ST239-*spa* t421 was identified as another clone circulating mainly in Portugal [42]. There were 99 MRSA isolates in our study classified as ST239, within which 92 isolates were ST239-*spa* t037 and 4 were ST239-*spa* t421. These findings indicated that those MRSA ST239 strains in Taiwan might be spread from Europe and America and disseminated islandwide.

Another clone, ST5 accounted for a minority of our MRSA collection (9 in 157 isolates, 5.7%), and eight of them were assigned as SCC*mec*II-*spa* t002. Nűbel et al investigated the evolution history of MRSA ST5 clone with SNPs mutation method and found that limited clades were identified but were not concordant with results of *spa* typing [43]. They also found that geographic spread of MRSA ST5 over a long distance was a rare event and the progeny of the ST5 resided locally rather than globally. The haplotype G, consisting of strains from East Asia including Taiwan, was mainly assigned as SCC*mec*II-*spa* t002. Our study displayed similar molecular typing results of MRSA ST5 strains with the haplotype G reported by Nűbel et al [43].

Based on polymorphisms of the *agr* gene, we classified our MRSA isolates into two groups (I and II), and most isolates (147, 93.6%) belonged to *agr* group I. This finding is consistent with that of Ho et al. [44] and Lu et al. [45]. Only 10 (6.4%) of our MRSA isolates belonged to *agr* group II. Previous studies have shown that some *agr* group II isolates are glycopeptide-intermediately resistant *S. aureus* (GISA) and hetero-GISA [46], [47]. In the study of Moise-Broder et al., *agr* group II isolates from 31 of 36 patients with treatment failure were found to be resistant to vancomycin [21]. In this study, no significant association was found between *agr* group II and vancomycin resistance.

Staphylococcal protein A (SpA) is a cell wall anchored virulence factor [48]. The polymorphic X region in the *spa* gene has been shown to be sufficiently stable for typing with a discriminatory power comparable to other typing methods including PFGE [16]. A total of 14 *spa* types were found in this study with types t037, t437, t002, and t421 being the majority. We also found that most *spa* t437 isolates belonged to SCC*mec*IV, SCC*mec*V_T_, and ST59, similar to the results of a previous studies on CA-MRSA [7], [49], [50]. In addition, most *spa* t037 isolates were found to belong to SCC*mec*III and ST239, and those of *spa* t002 were mostly SCC*mec*II and ST5. These results agree with those of HA-MRSA typing in other studies [7], [27], [51]. It has been reported that bacteremic patients in ICU and those with prolonged hospitalization were more likely to be infected with MRSA strains with elevated vancomycin MIC (2 mg/L), in which more than 80% of strains belonged to SCC*mec*III-*spa* t037 [27]. Our study also showed that SCC*mec*III-*spa* t037 (105 isolates, 66.9%) constituted most of the MRSA blood isolates with higher vancomycin MICs than those of CA-MRSA. This may provide a guide in empirical antibiotic therapy for MRSA bacteremia.

The increased vancomycin MIC of MRSA is a great concern. Two studies reveal that the success rates of glycopeptide treatment are decreased in MRSA bacteremic patients if vancomycin MIC of the MRSA isolate exceeds 1 mg/L [21], [22]. Wang et al. demonstrated that bacteremic patients in ICU and those with prolonged hospitalization were more likely to be infected with MRSA strains with elevated vancomycin MIC (2 mg/L), and those with elevated vancomycin MIC were associated with increased mortality rate [27]. In this study, vancomycin MICs of all MRSA blood isolates were found to be equal to or greater than 1 mg/L (range: 1–3 mg/L).

Daptomycin, a cyclic lipopeptide, was approved by the U.S. Food and Drugs Administration (FDA) for treatment of complicated skin and soft tissue infections, bacteremia, and endocarditis caused by *S. aureus*, including MRSA [52], [53]. Cui et al. had reported that reduced susceptibility of GISA to vancomycin and daptomycin might result from increased thickness of bacterial wall [54]. We found that elevated daptomycin MIC was associated with particular types such as SCC*mec*II and SCC*mec*III; ST5 and ST239; *spa* t002, t037 and t421; *dru*4, *dru*10, *dru*12, *dru*13, and *dru*14. These isolates also had elevated vancomycin MICs (Pearson's correlation coefficient *r* = 0.47) (Fig. 1). These findings may aid in the selection of appropriate antibiotic for MRSA and GISA infections.

Clindamycin has been recommended for non-critical infections caused by *S. aureus*, including MRSA [37], [55]. Resistance to clindamycin may be constitutive (MLSBc) or inducible (MLSBi) by erythromycin, and the latter may be misinterpreted as clindamycin susceptible by the traditional disc diffusion method. We found 13 isolates (8.3%) with the MLSBi phenotype, but they were not associated with any specific molecular types. The high resistance rate to erythromycin (94.9%) and clindamycin (86.5%) with low rate of MLSBi phenotype may represent a high percentage of our MRSA isolates with the MLSBc phenotype.

Panton-Valentine leukocidin (*pvl*) is associated with necrotizing pneumonia [23], scald skin syndrome [56], atopic dermatitis [57], wounds [56], bacteremia [56], and osteoarticular infections [58]. In this study, 13 MRSA isolates were found to harbor the *pvl* gene. Unlike the previous reports by Vandenesch et al. [59] and Udo et al. [60] in which most *pvl*+ MRSA isolates belonged to SCC*mec*IV, most of our *pvl*+ MRSA isolates were associated with SCC*mec*V_T_ (11 isolates, 84.6%), ST59 (12 isolates, 92.3%), *spa* t437 (11 isolates, 84.6%), and *dru*11 (8 isolates, 61.5%). These results resembled those of the previous report that the Taiwan clone (SCC*mec*V_T_-ST59-*spa* t437 or t441-*pvl*+) with resistance to erythromycin and tetracycline constituted most of the *pvl*+ CC59 CA-MRSA strains found in Western Australia [49].

*S. aureus* produces a number of toxins including staphylococcal enterotoxins (SEs) and toxic shock syndrome toxin-1 (TSST-1), which may cause food poisoning, enterocolitis, toxic shock syndrome, and autoimmune disease in human and livestock [24], [61]. More than 20 different SEs have been found. We found that HA-MRSA isolates (77% of SCC*mec*III and SCC*mec*II) harbored more SEs than CA-MRSA (15% of SCC*mec*IV and 7% of SCC*mec*V_T_). This result is similar to that of Tristan et al. [19]. It has been shown that each SCC*mec* type of MRSA contains a certain type of SE gene. For example, 77% of SCC*mec*II and 87% SCC*mec*III isolates are *sec*+, whereas 68% of SCC*mec*IV and 80% of SCC*mec*V isolates are *seb+* [26]. We also found that some SE genes were significantly associated with certain molecular types (for example, *sea*, *selk*, and *selq* with SCC*mec*III-ST59-*spa* t037; *seb* with SCC*mec*IV or V_T_-ST59-*spa* t437; and *tst-1* with SCC*mec*II-ST5-*agr*II-*spa* t002). Some isolates harbored multiple toxins, and a total of 15 superantigenic toxin gene profiles were found in our study. Interestingly, we found that some particular combination of toxin genes were associated with specific SCC*mec* types and *agr* groups (e.g., *sea*-*selk*-*selq* and *selk*-*selq* in SCC*mec*III-*agr*I, *seb*-*selk*-*selq* in SCC*mec*IV or V_T_-*agr*I, and *sec*-*seg*-*sei*-*sell*-*sel*m-*selp*-*tst1* in SCC*mec*II-*agr*II). This finding was different from that reported by Becker et al, in which 429 *S. aureus* isolates (219 blood isolates and 210 isolates from anterior nasal nares) from Germany were found to harbor specific toxic gene combinations such as *seg*-*sei* (55% of all isolates and 53% of blood isolates) as well as *sed*-*sej* (7% of all isolates and 10.5% of blood isolates) [62]. These findings indicated that MRSA blood isolates from Taiwan had unique toxin gene profiles, suggesting different geographical distribution of MRSA clones.

In conclusion, we have identified four major MRSA clone complexes CC1, CC5, CC8, and CC59 in Taiwan with the following molecular types: CC1: SCC*mec*IV-ST573; CC5: SCC*mec*II- ST5; CC8: SCC*mec*III-ST239 or ST241; and CC59: SCC*mec*IV or SCC*mec*V_T_-ST59 or ST338. The toxin gene profiles of these MRSA clone complexes are CC1: *sec*-*seg*-(*sei*)-*sell*-*sel*m-(*seln*)-*sel*o; CC5: *sec*-*seg*-*sei*-*sell*-*sel*m-(*seln*)-*selp*-*tst1*; CC8: *sea*-*selk*-*selq*, and CC59: *seb*-*selk*-*selq*. In addition, CC1 and CC5 clones had higher levels of vancomycin MIC than CC59. Continued surveillance of genotypes and phenotypes of MRSA isolates is needed to delineate the epidemiologic changes and to provide a positive impact on clinical practice.

### Ethics Statement

There was no human participant, patient data, or animal study included in this experimentation.

## Supporting Information

Table S1 **Relationship between various molecular types of 157 MRSA blood isolates.**
(DOC)

Table S2 **Distribution of various molecular types of 157 blood isolates among 9 medical centers in Taiwan.**
(DOC)

Table S3 **Distributions of superantigenic toxin genes in different molecular types of MRSA blood isolates.**
(DOC)
