# Supplementary material for: Molecular Typing and Phenotype Characterization of Methicillin-Resistant Staphylococcus aureus Isolates from Blood in Taiwan
Source: PLoS One. 2012 Jan 23;7(1):e30394. doi: 10.1371/journal.pone.0030394 (PMC3264593; doi:10.1371/journal.pone.0030394)
Supplement: Table S1 — Relationship between various molecular types of 157 MRSA blood isolates. (DOC) [file pone.0030394.s001.doc]

Table S1. Relationship between various molecular types of 157 MRSA blood isolates

| Molecular types | *agr* types | | Multilocus sequence type (MLST) | | | | | *spa* type | | | | | Copy no. of direct repeat unit (*dru*) | | | | | | | | | no. |
| --- | --- | --- | --- | --- | --- | --- | --- | --- | --- | --- | --- | --- | --- | --- | --- | --- | --- | --- | --- | --- | --- | --- |
|  | *agr*I | *agr*II | ST5 | ST239 | ST241 | ST59 | Others^1^ | t002 | t037 | t421 | t437 | Others | 4 | 6 | 9 | 10 | 11 | 12 | 13 | 14 | Others |  |
| SCC*mec*II | 3 | 6^***^ | 9^***^ |  |  |  |  | 8^***^ |  |  |  | 1 | 9^***^ |  |  |  |  |  |  |  |  | 9 |
| SCC*mec*III | 115^***^ |  |  | 99^***^ | 12^*^ |  | 4 |  | 105^***^ | 7 |  | 3 | 1 | 8 | 2 | 6 | 3 | 10 | 5 | 76^***^ | 4 | 115 |
| SCC*mec*IV | 17 | 4^*^ |  |  |  | 17^***^ | 4^***1^ |  | 1 |  | 11^***^ | 9 |  |  | 19^***^ |  |  |  |  |  | 2 | 21 |
| SCC*mec*V | 1 |  |  |  |  |  | 1 |  | 1 |  |  |  |  |  |  | 1 |  |  |  |  |  | 1 |
| SCC*mec*V_T_ | 11 |  |  |  |  | 10^***^ | 1 |  |  |  | 9^***^ | 2 |  |  | 2 |  | 8^***^ | 1 |  |  |  | 11 |
| *agr*I |  |  | 3 | 99^***^ | 12 | 26 | 7 | 3 | 106^***^ | 7 | 20 | 11 | 4 | 8 | 19 | 7 | 11 | 11 | 5 | 76^**^ | 6 | 147 |
| *agrII* |  |  | 6^***^ |  |  | 1 | 3^***1^ | 5^***^ | 1 |  |  | 4 | 6^***^ |  | 4^***^ |  |  |  |  |  |  | 10 |
| ST5 |  |  |  |  |  |  |  | 8^***^ |  |  |  | 1 | 9^***^ |  |  |  |  |  |  |  |  | 9 |
| ST239 |  |  |  |  |  |  |  |  | 92^***^ | 4 |  | 3 |  |  | 2 | 6 | 3 | 10^*^ | 5 | 72^***^ | 1 | 99 |
| ST241 |  |  |  |  |  |  |  |  | 9 | 3^***^ |  |  | 1 | 8^***^ |  |  |  |  |  |  | 3 | 12 |
| ST59 |  |  |  |  |  |  |  |  |  |  | 20^***^ | 7 |  |  | 17^***^ |  | 7^***^ | 1 |  |  | 2 | 27 |
| other ST |  |  |  |  |  |  |  |  | 6 |  |  | 4 |  |  | 4^***1^ | 1 | 1 |  |  | 4 |  | 10 |
| *spa* t002 |  |  |  |  |  |  |  |  |  |  |  |  | 8^***^ |  |  |  |  |  |  |  |  | 8 |
| *spa* t037 |  |  |  |  |  |  |  |  |  |  |  |  | 1 | 5 | 3 | 7 | 2 | 9 | 4 | 72^***^ | 4 | 107 |
| *spa* t421 |  |  |  |  |  |  |  |  |  |  |  |  |  | 3^***^ |  |  |  |  | 1 | 3 |  | 7 |
| *spa* t437 |  |  |  |  |  |  |  |  |  |  |  |  |  |  | 11^***^ |  | 6^***^ | 1 |  |  | 2 | 20 |
| other *spa* |  |  |  |  |  |  |  |  |  |  |  |  | 1 |  | 9 |  | 3 | 1 |  | 1 |  | 15 |
| Total no. | 147 | 10 | 9 | 99 | 12 | 27 | 10 | 8 | 107 | 7 | 20 | 15 | 10 | 8 | 23 | 7 | 11 | 11 | 5 | 76 | 6 | 157 |

^*^*p* <0.05, ^**^ *p* <0.01, ^***^ *p* <0.001; ^1^ for ST573
