# Supplementary material for: Molecular Typing and Phenotype Characterization of Methicillin-Resistant Staphylococcus aureus Isolates from Blood in Taiwan
Source: PLoS One. 2012 Jan 23;7(1):e30394. doi: 10.1371/journal.pone.0030394 (PMC3264593; doi:10.1371/journal.pone.0030394)
Supplement: Table S2 — Distribution of various molecular types of 157 blood isolates among 9 medical centers in Taiwan. (DOC) [file pone.0030394.s002.doc]

Table S2. Distribution of various molecular types of 157 blood isolates

among 9 medical centers in Taiwan

| Major types/ Hospitals | N1 | N2 | N3 | C1 | C2 | C3 | S1 | S3 | E1 | Total |
| --- | --- | --- | --- | --- | --- | --- | --- | --- | --- | --- |
| ST5 | 2 | 1 | 0 | 1 | 0 | 3 | 1 | 0 | 1 | 9 |
| ST239 | 39 | 3 | 11 | 1 | 1 | 10 | 19 | 1 | 14 | 99 |
| ST241 | 6 | 0 | 3 | 1 | 0 | 1 | 1 | 0 | 0 | 12 |
| ST59 | 16 | 0 | 3 | 0 | 0 | 3 | 1 | 0 | 4 | 27 |
| ST573 | 4 | 0 | 0 | 0 | 0 | 0 | 0 | 0 | 0 | 4 |
| ST900 | 2 | 0 | 0 | 0 | 0 | 0 | 0 | 0 | 0 | 2 |
| Other ST | 1 | 0 | 0 | 0 | 0 | 1 | 2 | 0 | 0 | 4 |
| *agr* I | 65 | 3 | 17 | 2 | 1 | 16 | 23 | 1 | 19 | 147 |
| *agr* II | 5 | 1 | 0 | 1 | 0 | 2 | 1 | 0 | 0 | 10 |
| SCC*mec*II | 2 | 1 | 0 | 1 | 0 | 3 | 1 | 0 | 1 | 9 |
| SCC*mec*III | 48 | 3 | 14 | 2 | 1 | 11 | 21 | 1 | 14 | 115 |
| SCC*mec*IV | 13 | 0 | 2 | 0 | 0 | 2 | 1 | 0 | 3 | 21 |
| SCC*mec*V | 0 | 0 | 1 | 0 | 0 | 1 | 0 | 0 | 0 | 1 |
| SCC*mec*V_T_ | 7 | 0 | 1 | 0 | 0 | 1 | 1 | 0 | 1 | 11 |
| *spa* t002 | 2 | 0 | 0 | 1 | 0 | 3 | 0 | 0 | 1 | 8 |
| *spa* t437 | 11 | 1 | 3 | 0 | 0 | 1 | 1 | 0 | 4 | 20 |
| *spa* t037 | 41 | 3 | 14 | 2 | 1 | 11 | 21 | 1 | 13 | 107 |
| *spa* t421 | 6 | 0 | 0 | 0 | 0 | 1 | 0 | 0 | 0 | 7 |
| Other *spa* types | 10 | 0 | 0 | 0 | 0 | 2 | 2 | 0 | 1 | 15 |

N: northern (3 hospitals including N1, N2, and N3); C: central (3 hospitals

including C1, C2, and C3); S: southern (2 hospitals including S1and S2);

and E: eastern (1 hospital, E1)
