# Supplementary material for: Molecular Typing and Phenotype Characterization of Methicillin-Resistant Staphylococcus aureus Isolates from Blood in Taiwan
Source: PLoS One. 2012 Jan 23;7(1):e30394. doi: 10.1371/journal.pone.0030394 (PMC3264593; doi:10.1371/journal.pone.0030394)
Supplement: Table S3 — Distributions of superantigenic toxin genes in different molecular types of MRSA blood isolates. (DOC) [file pone.0030394.s003.doc]

Table S3. Distributions of superantigenic toxin genes in different molecular types of MRSA blood isolates

| Toxin genes | SCC*mec* type no. (%) | | | | | *agr* type no. (%) | | no. (%) |
| --- | --- | --- | --- | --- | --- | --- | --- | --- |
|  | Ⅱ | Ⅲ | Ⅳ | Ⅴ | Ⅴ_T_ | Ⅰ | Ⅱ |  |
| *sea* | 0(0) | 103(66) ^***^ | 1(1) | 1(1) | 0(0) | 104(66) ^***^ | 1(1) | 105(67) |
| *seb* | 0(0) | 1(1) | 17(11) ^***^ | 0(0) | 8(5) ^***^ | 25(16) | 1(1) | 26(17) |
| *sec* | 6(4)^***^ | 0(0) | 6(4) ^***^ | 0(0) | 0(0) | 4(3) | 8(5) ^***^ | 12(8) |
| *sed* | 4(0) | 0(0) | 0(0) | 0(0) | 0(0) | 0(0) | 1(1) | 1(1) |
| *sS0000000000000000000000000000000000000000000000000000000000000000000000000000000000000000000000000000000000000000000000000000000ee* | 0(1) | 0(0) | 0(0) | 0(0) | 0(0) | 0(0) | 0(0) | 0(0) |
| *seg* | 7(4) ^***^ | 0(0) | 6(4) ^***^ | 0(0) | 0(0) | 4(3) | 9(6) ^***^ | 13(8) |
| *seh* | 0(0) | 0(0) | 0(0) | 0(0) | 0(0) | 0(0) | 0(0) | 0(0) |
| *sei* | 7(4) ^***^ | 0(0) | 7(4) ^***^ | 0(0) | 0(0) | 5(3) | 9(6) ^***^ | 14(9) |
| *selj* | 0(0) | 0(0) | 0(0) | 0(0) | 0(0) | 0(0) | 0(0) | 0(0) |
| *selk* | 0(0) | 108(69) ^***^ | 17(11) ^***^ | 1(1) | 8(5) ^***^ | 133(85) ^***^ | 1(1) | 134(85) |
| *sell* | 7(4) ^***^ | 0(0) | 6(4) ^**^ | 1(1) | 0(0) | 5(3) | 9(6) ^***^ | 14(9) |
| *selm* | 7(4) ^***^ | 0(0) | 6(4) ^***^ | 0(0) | 0(0) | 4(3) | 9(6) ^***^ | 13(8) |
| *seln* | 3(2) | 0(0) | 3(2) ^**^ | 0(0) | 0(0) | 3(2) | 3(2) ^***^ | 6(4) |
| *selo* | 7(4) ^***^ | 0(0) | 6(4) ^***^ | 0(0) | 0(0) | 4(3) | 9(6) ^***^ | 13(8) |
| *selp* | 7(4) ^***^ | 0(0) | 8(5) ^***^ | 0(0) | 0(0) | 9(6) | 6(4) ^***^ | 15(10) |
| *selq* | 0(0) | 108(69) ^***^ | 17(11) ^***^ | 0(0) | 8(5) ^***^ | 132(85) ^***^ | 1(1) | 133(85) |
| *selr* | 0(0) | 0(0) | 0(0) | 0(0) | 0(0) | 0(0) | 0(0) | 0(0) |
| *tst1* | 7(4) ^***^ | 0(0) | 2(1) | 0(0) | 0(0) | 3(2) | 6(4) ^***^ | 9(6) |
| Total | 7(4) | 115(73) | 23(15) | 1(1) | 11(7) | 147(94) | 10(6) | 157(100) |

Table S3. (Continued)

| Toxin genes | Multilocus sequence type no.(%) | | | | | *spa* type no.(%) | | | | | Copy no. of direct repeat units no.(%) | | | | | | | | | no. (%) |
| --- | --- | --- | --- | --- | --- | --- | --- | --- | --- | --- | --- | --- | --- | --- | --- | --- | --- | --- | --- | --- |
|  | ST5 | ST239 | ST241 | ST59 | others | t002 | t037 | t421 | t437 | others | 4 | 6 | 9 | 10 | 11 | 12 | 13 | 14 | others |  |
| *sea* | 1(1) | 97(62) ^***^ | 1(1) | 1(1) | 5(3) | 1(1) | 96(61) ^***^ | 4(3) | 1(1) | 3(2) | 1(1) | 0(0) | 2(0) | 7(4) | 3(2) | 10(6) | 5(3) | 75(48)^***^ | 2(1) | 105(67) |
| *seb* | 0(0) | 1(1) | 0(0) | 24(15) ^***^ | 1(1) | 0(0) | 1(1) | 0(0) | 17(11) | 8(5) | 0(0) | 0(0) | 16(10) ^***^ | 0(0) | 7(4) ^***^ | 1(1) | 0(0) | 0(0) | 2(1) | 26(17) |
| *sec* | 8(5) ^***^ | 0(0) | 0(0) | 0(0) | 4(3) ^***1^ | 7(4) ^***^ | 1(1) | 0(0) | 0(0) | 4(3) | 8(5) ^***^ | 0(0) | 4(3) | 0(0) | 0(0) | 0(0) | 0(0) | 0(0) | 0(0) | 12(8) |
| *sed* | 1(1) | 0(0) | 0(0) | 0(0) | 0(0) | 1(1) | 0(0) | 0(0) | 0(0) | 0(0) | 1(1) | 0(0) | 0(0) | 0(0) | 0(0) | 0(0) | 0(0) | 0(0) | 0(0) | 1(1) |
| *see* | 0(0) | 0(0) | 0(0) | 0(0) | 0(0) | 0(0) | 0(0) | 0(0) | 0(0) | 0(0) | 0(0) | 0(0) | 0(0) | 0(0) | 0(0) | 0(0) | 0(0) | 0(0) | 0(0) | 0(0) |
| *seg* | 9(6) ^***^ | 0(0) | 0(0) | 0(0) | 4(3) ^***1^ | 8(5) ^***^ | 1(1) | 0(0) | 0(0) | 4(3) | 9(6) ^***^ | 0(0) | 4(3) | 0(0) | 0(0) | 0(0) | 0(0) | 0(0) | 0(0) | 13(8) |
| *seh* | 0(0) | 0(0) | 0(0) | 0(0) | 0(0) | 0(0) | 0(0) | 0(0) | 0(0) | 0(0) | 0(0) | 0(0) | 0(0) | 0(0) | 0(0) | 0(0) | 0(0) | 0(0) | 0(0) | 0(0) |
| *sei* | 9(6) ^***^ | 0(0) | 0(0) | 2(1) | 3(2) ^*1^ | 8(5) ^***^ | 1(1) | 0(0) | 0(0) | 3(2) | 9(6) ^***^ | 0(0) | 5(3) ^*^ | 0(0) | 0(0) | 0(0) | 0(0) | 0(0) | 0(0) | 14(9) |
| *selj* | 0(0) | 0(0) | 0(0) | 0(0) | 0(0) | 0(0) | 0(0) | 0(0) | 0(0) | 0(0) | 0(0) | 0(0) | 0(0) | 0(0) | 0(0) | 0(0) | 0(0) | 0(0) | 0(0) | 0(0) |
| *selk* | 0(0) | 93(59) ^***^ | 12(8) | 24(15) | 5(3) | 0(0) | 99(63) ^***^ | 7(4) | 17(11) | 11(7) ^***2^ | 1(1) | 8(5) | 17(11) | 7(4) | 10(6) | 11(7) | 5(3) | 69(44) | 6(4) | 134(85) |
| *sell* | 9(6) ^***^ | 0(0) | 0(0) | 0(0) | 5(3) ^***1^ | 8(5) ^***^ | 2(1) | 0(0) | 0(0) | 4(3) | 9(6) ^***^ | 0(0) | 4(3) | 1(1) | 0(0) | 0(0) | 0(0) | 0(0) | 0(0) | 14(9) |
| *selm* | 9(6) ^***^ | 0(0) | 0(0) | 0(0) | 4(3) ^***1^ | 8(5) ^***^ | 1(1) | 0(0) | 0(0) | 4(3) | 9(6) ^***^ | 0(0) | 4(3) | 0(0) | 0(0) | 0(0) | 0(0) | 0(0) | 0(0) | 13(8) |
| *seln* | 4(3) ^***^ | 0(0) | 0(0) | 0(0) | 2(1) ^***1^ | 4(3) ^***^ | 0(0) | 0(0) | 0(0) | 2(1)^*3^ | 4(3) ^***^ | 0(0) | 2(1) | 0(0) | 0(0) | 0(0) | 0(0) | 0(0) | 0(0) | 6(4) |
| *selo* | 9(6) ^***^ | 0(0) | 0(0) | 0(0) | 4(3) ^***1^ | 8(5) ^***^ | 1(1) | 0(0) | 0(0) | 4(3) | 9(6) ^***^ | 0(0) | 4(3) | 0(0) | 0(0) | 0(0) | 0(0) | 0(0) | 0(0) | 13(8) |
| *selp* | 9(6) ^***^ | 1(1) | 0(0) | 5(3) | 0(0) | 8(5) ^***^ | 1(1) | 0(0) | 4(0) | 2(1) | 9(6) ^***^ | 0(0) | 4(3) | 0(0) | 0(0) | 0(0) | 0(0) | 0(0) | 0(0) | 15(10) |
| *selq* | 0(0) | 93(59) ^***^ | 12(8) | 24(15) | 4(3) | 0(0) | 98(62) ^***^ | 7(4) | 17(11) | 11(7) ^***2^ | 1(1) | 8(5) | 17(11) | 6(4) | 10(6) | 11(7) | 5(3) | 69(44) ^*^ | 6(4) | 133(85) |
| *selr* | 0(0) | 0(0) | 0(0) | 0(0) | 0(0) | 0(0) | 0(0) | 0(0) | 0(0) | 0(0) | 0(0) | 0(0) | 0(0) | 0(0) | 0(0) | 0(0) | 0(0) | 0(0) | 0(0) | 0(0) |
| *tst1* | 9(6) ^***^ | 0(0) | 0(0) | 0(0) | 0(0) | 8(5) ^***^ | 0(0) | 0(0) | 0(0) | 1(1) | 9(6) ^***^ | 0(0) | 0(0) | 0(0) | 0(0) | 0(0) | 0(0) | 0(0) | 0(0) | 9(6) |
| No. of isolates | 9(6) | 99(63) | 12(8) | 27(17) | 10(6) | 8(5) | 107(68) | 7(4) | 20(13) | 15(10) | 10(6) | 8(5) | 23(15) | 7(4) | 11(7) | 11(7) | 5(3) | 76(48) | 6(4) | 157(100) |

^*^*p* <0.05, ^**^ *p* <0.01, ^***^ *p* <0.001, ^1^ for ST573；^2^ for *spa* t3592；^3^ for *spa* t3406
